# A meta-review of stress, coping and interventions in dementia and dementia caregiving

**DOI:** 10.1186/s12877-016-0280-8

**Published:** 2016-05-18

**Authors:** K. J. Gilhooly, M. L. M. Gilhooly, M. P. Sullivan, A. McIntyre, L. Wilson, E. Harding, R. Woodbridge, S. Crutch

**Affiliations:** Department of Clinical Sciences, Brunel University London, Uxbridge, UB8 3PH UK; Dementia Research Centre, Institute of Neurology, University College London, London, WC1N 3BG UK

**Keywords:** Dementia, Caregiver, Stress, Coping, Interventions, Systematic review, Meta-review

## Abstract

**Background:**

There has been a substantial number of systematic reviews of stress, coping and interventions for people with dementia and their caregivers. This paper provides a meta-review of this literature 1988-2014.

**Method:**

A meta-review was carried out of systematic reviews of stress, coping and interventions for people with dementia and their caregivers, using SCOPUS, Google Scholar and CINAHL Plus databases and manual searches.

**Results:**

The meta-review identified 45 systematic reviews, of which 15 were meta-analyses. Thirty one reviews addressed the effects of interventions and 14 addressed the results of correlational studies of factors associated with stress and coping. Of the 31 systematic reviews dealing with intervention studies, 22 focused on caregivers, 6 focused on people with dementia and 3 addressed both groups. Overall, benefits in terms of psychological measures of mental health and depression were generally found for the use of problem focused coping strategies and acceptance and social-emotional support coping strategies. Poor outcomes were associated with wishful thinking, denial, and avoidance coping strategies. The interventions addressed in the systematic reviews were extremely varied and encompassed Psychosocial, Psychoeducational, Technical, Therapy, Support Groups and Multicomponent interventions. Specific outcome measures used in the primary sources covered by the systematic reviews were also extremely varied but could be grouped into three dimensions, *viz*., a broad dimension of “Psychological Well-Being v. Psychological Morbidity” and two narrower dimensions of “Knowledge and Coping” and of “Institutionalisation Delay”.

**Conclusions:**

This meta-review supports the conclusion that being a caregiver for people with dementia is associated with psychological stress and physical ill-health. Benefits in terms of mental health and depression were generally found for caregiver coping strategies involving problem focus, acceptance and social-emotional support. Negative outcomes for caregivers were associated with wishful thinking, denial and avoidance coping strategies. Psychosocial and Psychoeducational interventions were beneficial for caregivers and for people with dementia. Support groups, Multicomponent interventions and Joint Engagements by both caregivers and people with dementia were generally found to be beneficial. It was notable that virtually all reviews addressed very general coping strategies for stress broadly considered, rather than in terms of specific remedies for specific sources of stress. Investigation of specific stressors and remedies would seem to be a useful area for future research.

**Electronic supplementary material:**

The online version of this article (doi:10.1186/s12877-016-0280-8) contains supplementary material, which is available to authorized users.

## Background

There has been extensive research over recent decades [1*, 2*, 3*] on the stressors experienced by people with dementia (PWD) and their informal caregivers (CGs). This research has explored the levels of stress experienced by CGs and PWD, the correlates of stress, coping strategies, and the benefits of a range of interventions. In terms of interventions, the present paper focuses on non-pharmacological interventions. As an indication of the volume of primary research in the area, it may be noted that an initial search of SCOPUS in September 2014, with terms (Alz* AND stress*) since 1988, produced 3537 titles. Excluding those primarily neurological, biochemical, pharmaceutical and medical, left 124 studies associated with caregiver stress. A search with (Alz* AND *coping*) led to 409 papers and a search with (Alz* AND careg*) produced 3739 titles. As part of a wider study on caregivers of people with dementia, we set out to examine the knowledge base in relation to factors affecting stress and coping in CGs and PWD and in relation to non-pharmacological interventions that have been found to be beneficial, in terms of reducing stress, increasing coping and improving the quality of life, for both CGs and PWD.

Examining the reference lists of highly cited primary research papers in the area suggested that there were a substantial number of *systematic reviews* of caregiver stress and coping. When a large volume of systematic reviews are available, as in the case of stress and coping in dementia caregiving, a higher level of synthesis is needed to synthesise and summarise findings. The present paper aims to provide such a high level synthesis of the information contained in the relevant systematic review literature, by means of a *meta-review* [4, 5].

## Method

A PRISMA 2009 Checklist is provided as a supplementary document accompanying this paper (Additional file 1).

### Search strategy

Electronic searches were carried out using SCOPUS, Google Scholar and CINAHL Plus databases, to identify relevant systematic reviews published in peer reviewed English language journals from January 1988 to December 2014. The earliest systematic review that we were aware of, before carrying out the full search reported here, was by Knight et al. [1*], and we extended the search period for a further 5 years from 1993 back to 1988 to capture possible earlier reviews. The search terms were (Alz* OR *dementia*) AND caregiv* AND (stress* OR *coping*) AND (*systematic review* OR meta*). In addition, the reference lists of articles identified by electronic searching were manually searched for further systematic reviews.

### Study selection

#### Inclusion criteria

Systematic reviews with a defined search strategy that attempted to quantitatively or qualitatively analyse primary studies. Both reviews with results pooled statistically in a meta-analysis and those with qualitative analyses were eligible. The inclusion criteria of the systematic reviews entering into the meta-review must have addressed stress in PWDs or CGs and non-pharmacological interventions for stress or correlates of stress.

#### Exclusion criteria

Non-English language, not peer-reviewed, editorials, correspondence, conference abstracts, and review summary papers.

The studies identified in the search were initially screened for relevance by one reviewer (KJG) on the basis of their titles and abstracts. Subsequently, two reviewers (KJG and MLMG) assessed the potentially relevant studies and agreed the studies selected on the bases of the eligibility criteria. Disagreements were resolved by consensus.

## Results

The initial electronic search process identified 93 candidate systematic review papers; of these, 47 were excluded as having a predominantly neurological, biochemical, or pharmaceutical focus and so were not relevant to our concern with non-pharmacological approaches or otherwise met the exclusion criteria.

The 46 relevant systematic reviews identified by our search procedure were given a quality rating using the Assessment of Multiple Systematic Reviews (AMSTAR) scoring scheme developed by Shea et al. [6] to assess the methodological quality of systematic reviews. The rating is based on an 11 item checklist. Total ratings have been found to have high inter-observer reliability (Intra-Class correlation = 0.84) and good construct validity [7]. One paper was excluded at this stage as having an unacceptably low AMSTAR rating (0).

**Fig. 1:**
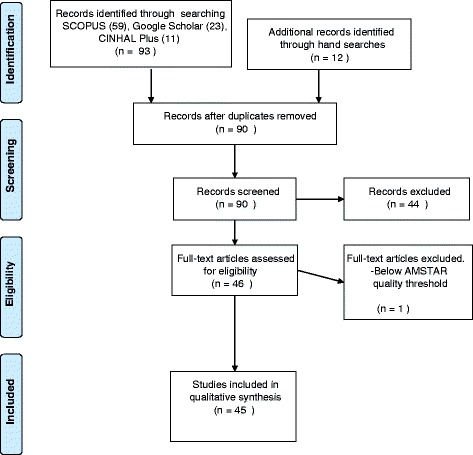
Flowchart of record identification and selection process

The review process is summarized in Fig. 1.

The 45 systematic review papers included in our analysis are summarised in Additional file 2, which shows AMSTAR quality rating, authorship, year of publication, the focus of each paper, the search strategy used by each review, whether or not a meta-analysis was conducted, the number of papers included or excluded in each review, and the conclusions drawn from each review.

The reviews in Additional file 2 range in date from 1993 to 2014 with a median publication year of 2007. The typical (median) systematic review searched 4 databases (range 1-25) with 8 keywords (range 2-26), included 25 studies (range 4-305), excluded 497 studies (range, 2-7402) and had a selection ratio (total included/(total included + total excluded) of 7 % (range < 1 % to 75 %). The AMSTAR ratings had a median of 6 and ranged from 2-9. AMSTAR quality ratings were significantly more positive for meta-analyses than for systematic reviews (*r* (44) = .42, *p* = .003), but were not related to year of publication, searching more databases, number of keywords used, number of papers included or excluded, or to selection ratios. There was a significant tendency for more recent reviews to be more selective in terms of the ratio of included papers (*r* (44) = -.38, *p* = .005, between year of publication and selection ratio).

An issue with reviewing reviews is that reviews overlap with each other in terms of the primary sources that go into the reviews. We examined overlap in a 50 % sample of reviews (*n* = 22). The method for checking overlap is labour intensive, hence the use of a sample in this analysis. To carry out the analysis, a table was drawn up manually of the primary sources used in the sample of 22 reviews, such that primary sources were the rows and the columns were the reviews. This initial process cannot be readily automated. An entry of “1” in the table of primary sources X reviews indicated that a given source was in a given review and an entry of “0” indicated that a given source was not in a given review. Over the 22 sampled reviews, there was a total of 805 sources, summing over the reviews, of which 322 were unique. This implies an overlap index of 7.2 % (using Pieper et al.’s “Corrected covered area” index [8], which ranges from 0 -100 %). An index of 6-10 % is classed as a moderate degree of overlap and the value obtained here (7.2 %) is similar to the value of 6.4 % found in Pieper et al.’s sample of 45 reviews of systematic reviews in medicine [8]. Clearly, some overlap over reviews would be expected as all the reviews are addressing the same general area of caregiver stress and coping in dealing with Alzheimer’s disease. However, the degree of overlap is within acceptable limits, as different reviews vary in the time-frames they cover and in search terms, exact issues addressed and professional audiences targeted.

The systematic reviews in Additional file 2 may be conveniently divided into those focusing on (a) Interventions involving Factorial Manipulation studies (*n* = 31, 70 %) and (b) Correlations/Associations as found in survey based studies (*n* = 14, 30 %).

Within the Intervention group, themes emerged with foci on CGs (*n* = 22), PWDs (*n* = 6) and both CGs and PWD (*n* = 3). See Table 1 for a list of reviews of intervention studies classified by focus.

**Table 1:** Intervention reviews classified by focus

Study Focus		
Caregivers	People with Dementia	Both CG and PWD
Knight, Lutzky, Macofsky-Urban (1993) [1*]	Zabalegui, Hamers, Karlsson *et al.* (2014) [3*]	Brodaty & Arasaratnam (2012) [10*]
Brodaty, Green, Koschera (2003) [2*]	Bates,Boote & Beverley (2003) [9*]	Olazaran, Reisberg, Clare *et al.*(2010) [13*]
Thinnes & Padilla (2011) [15*]	Hogan, Bailey, Carswell *et al.* (2007) [11*]	Smits, de Lange, Droes *et al.*. (2007) [16*]
Cooper, Balamuri, Selwood *et al.*(2007) [37*]	O’Connor, Ames, Gardner *et al.* (2009) [12*]	
Thompson, Spilsbury, Hall *et al.* (2007) [21*]	Spiker, Vernooij-Dassen, Vasse *et al.* (2008) [14*]	
Pusey & Richards (2001) [29*]	Cooper, Mukadam, Katona *et al.* (2012) [38*]	
Li, Cooper, Austin *et al.* (2013) [30*]		
Cooke, McNally, Mulligan *et al.*(2001) [33*]		
Pinquart & Sorensen (2006) [34*]		
Acton & Kang (2001) [35*]		
Chien, Chu, Guo *et al.* (2011) [36*]		
Elvish, Lever, Johnstone *et al.* (2013) [39*]		
Hall & Skelton (2011) [41*]		
LoGuidice & Hassett (2005) [42*]		
Parker, Mills & Abbey (2008) [44*]		
Peacock & Forbes (2003) [45*]		
Powell, Chiu, & Eysenbach (2008) [46*]		
Schoenmakers, Buntinx & deLepeleire(2010) [48*]		
Schulz, O’Brien, Czaja *et al* (2002). [49*]		
Selwood, Johnston, Katona *et al.* (2007) [50*]		
Sorensen, Pinquart & Duberstein (2002) [51*]		
Vernooij-Dassen, Draskovic, McCleery *et al.* (2011) [53*]		

*CG* Caregiver focus, *PWD* Person with Dementia focus; Both = CG and PWD addressed

Within the Correlation/Association grouping, reviews could be subdivided into those focusing on associations between (a) PWD symptoms and CG stress (*n* = 3), (b) CG characteristics and CG stress (*n* = 6), and (c) coping strategies and CG stress (*n* = 5). See Table 2 for a list of reviews of correlational studies classified by focus.

**Table 2:** Correlation/association based reviews classified by focus

	Focus	
PWD symptoms – CG stress	CG characteristics – CG stress	Coping – CG stress
Black & Almeida (2004) [22*]	Cooper, Balamurali & Livingston (2007) [17*]	Gottlieb & Wolfe (2002) [27*]
Ornstein & Guagler (2012) [25*]	Cuijpers (2005) [18*]	Kneebone & Martin (2003). [28*]
Luppa, Luck, Brahler *et al.* (2008) [43*]	Pinquart & Sorensen (2007) [19*]	Li, Cooper, Bradeley *et al.* (2012). [31*]
	Vitaliano, Zhang & Scanlan (2003) [20*]	Del-Pino-Casada, Frias-Osuna, Palomino-Moral *et al.* (2011) [32*]
	Lee, Bakker, Duivenvoorden *et al.* (2014) [21*]	Quinn, Clare & Woods (2010) [47*]
	Etters, Goodall & Harrison (2008) [40*]	

*PWD* Person with dementia, *CG* Caregiver

The interventions discussed in the reviews were extremely varied. Overall, 20 distinct interventions appear over the abstracts of the 32 reviews that focused on interventions. The terms used in the intervention reviews could be grouped into six categories: Psychosocial (*n* = 5), Psychoeducational (*n* =7), Technical (*n* = 5), Therapy (*n* = 6), Support Groups (*n* = 3) and Multicomponent (*n* = 5) interventions.

Specific outcome measures over the primary sources covered by the reviews were extremely varied, covering burden, stress, anxiety, depression, well-being *etc*. The outcome measures could mostly be grouped into a single broad dimension of “Psychological Well-Being v. Psychological Morbidity”, and into two narrower dimensions of “Knowledge, Behaviour and Coping”, and “Institutionalisation Delay”.

For PWD there was evidence of benefits in terms of behavior and coping from psychosocial and psychoeducational interventions [9*, 10*, 11*, 12*]. Delays to institutionalisation were found with support programs, psychosocial and multicomponent interventions [3*, 13*, 14*].

The principal results relating CG focused interventions to the major outcomes (CG Wellbeing and CG Knowledge and Coping) are indicated in Table 3. From Table 3, it appears that benefits for CGs accrued from both psychosocial and psychoeducational interventions. Support groups were also useful. Multicomponent interventions were generally helpful and joint engagements by CG and PWD in Psychosocial and Psychoeducational interventions were also found to beneficial [15*, 16*].

**Table 3:** CG related interventions by explicitly reported outcomes

Outcomes				
	Psychological Wellbeing		Knowledge/Coping	
Intervention	Positive benefit	No benefit	Positive benefit	No benefit
Psychosocial	7	1	2	-
	Knight *et al.*, 1993 [1*]	Schoenmakers *et al.*, 2010 [48*]	Brodaty *et al.*, 2003 [10*]	
	Brodaty *et al.*, 2012. [2*]		Pusey & Richards, (2001). [29*]	
	Brodaty *et al.*, 2003 [10*]			
	O’Connor *et al.* 2009. [12*]			
	Cooper *et al.*, 2007. [17*]			
	Schulz *et al.*, (2002). [49*]			
	Thompson *et al.*, 2007 [52*]			
Psychoeducational	6	2	1	1
	Hogan *et al.*, 2007. [11*]	Acton & Kang, 2001. [35*]	Elvish *et al.*, 2013. [39*]	Selwood *et al.*, 2007. [50*]
	Cooke *et al.*, 2001. [33*]	Selwood *et al.*, 2007. [50*]		
	Pinquart & Sorensen, 2006. [34*]			
	Elvish *et al.*, 2013. [39*]			
	Parker *et al.*, 2008. [44*]			
	Sorensen *et al.*, 2002. [51*]			
Technological	1	-	-	1
	Powell *et al.*, 2008 [46*]			Peacock & Forbes, 2003. [45*]
Therapy	4	1	1	1
	Zabalegui *et al.*, 2014. [3*]	Acton & Kang, 2001 [35*]	Selwood *et al.*, 2007 [50*]	Peacock & Forbes, 2003. [45*]
	Hall & Skelton, 2011. [41*]			
	Selwood *et al.*, 2007. [50*]			
	Vernooij-Dassen, 2011. [53*]			
Social Support	5	1	4	-
	Hogan *et al.*, 2007. [11*]	Knight *et al.*, 1993. [1*]	Zabalegui *et al.*, 2014 [3*]	
	Spijker *et al.*, 2008. [14*]		Spijker *et al.*, 2008. [14*]	
	Gottlieb & Wolfe, 2002. [27*]		Thinnes & Padilla, 2011. [15*]	
	Cooke *et al.*, 2001. [33*]		Li *et al.*, 2013. [30*]	
	Chien *et al.*, 2011 [36*]			
Multi-component	6	-	3	1
	Smits et al, 2007. [16*]		Olazaran *et al.*, 2010 [13*]	Selwood *et al.*, 2007. [50*]
	Cooke *et al.*, 2001 [33*]		Elvish *et al.*, 2013. [39*]	
	Acton & Kang, 2001. [35*]		Selwood *et al.*, 2007. [50*]	
	Elvish *et al.*, 2013. [39*]			
	Etters *et al.*, 2008. [40*]			
	Parker *et al.*, 2008. [44*]			
Totals	29	5	11	4

## Discussion

There was clear support from the systematic reviews for the view that being a CG for PWD is a risk factor for psychological stress [17*, 18*] and physical ill-health [19*, 20*]. Although PWD’s behavioural problems impact strongly on CG stress [21*, 22*], nearly all the studies included in the various reviews listed here dealt with very general coping strategies and with interventions for general “stress” associated with caring, without delving into specific stressors (such as wandering, memory problems, visual problems, aggressiveness etc.) or specific remedies, such as improved lighting to assist with visual problems [23, 24]. An exception is Ornstein and Gaugler’s review [25*] which pinpoints particularly troubling symptoms in Alzheimer’s disease, namely depression, aggression and sleep disturbance that negatively impact on CGs; however, they do not identify specific remedies. Consideration of specific stressors and remedies would seem to be a useful area for development for future research.

Many of the correlational studies reviewed in the systematic reviews used the Lazarus & Folkman model of coping [26], where coping strategies are of two main types: (1) *emotion-focused* - *e.g*., avoidance, minimising, seeking positive value in negative events and (2) *problem-focused* - *e.g*., defining the problem, seeking alternative solutions, choosing and acting. Overall, benefits in terms of mental health and depression were generally found for problem-focused coping, acceptance and social-emotional support [15*, 27*, 28*, 29*, 30*]. Wishful thinking, denial, and avoidance strategies were found to be associated with negative outcomes [27*, 31*, 32*].

Although the terms “psychosocial intervention” and “psychoeducational intervention” appeared in 12 out of 45 reviews these terms were rarely explicitly defined in the reviews. It would, however, seem useful to consider how these terms might be defined. In earlier studies [29*, 33*], the terms “psychosocial” and “psychoeducational” are used rather interchangeably. For example, Pusey & Richards [29*] define psychosocial interventions as “…interpersonal interventions concerned with the provision of information, education, or emotional support together with individual psychological interventions addressing a specific health and social care outcome”, and so include education within psychosocial interventions. Cooke et al. [33*s] listed fifteen types of intervention as “psychosocial”, including predominantly educational interventions such as general education, social skills training, cognitive problem solving and practical caregiving skills, as well as predominantly psychological interventions such as cognitive therapy, relaxation, psychotherapy and counselling. However, Pinquart & Sorensen [34*] explicitly define psychoeducational interventions as involving a “…focus on the structured presentation of information about dementia and caregiving-related issues and may include an active role of participants (e.g., role playing, applying new knowledge to individual problems).” In terms of the Lazarus-Folkman coping model [26], it can be argued that prototypical psychoeducational interventions aim at the development of problem-focused coping strategies while prototypical psychosocial interventions address the development of emotion-focused coping strategies.

### Strengths and limitations

The systematic reviews contributing to the present meta-review draw on 1,900 citations and range over 20 years of systematic reviews (1993-2014) and so the conclusions reached in this paper have an extensive empirical base and reflect recent as well as longer established findings.

A problem with reviews of reviews is that reviews overlap in terms of their primary sources as all reviews are addressing the same broad area, in this case, CG stress and coping. This means that highly cited primary papers that appear in many reviews could over-weight the apparent evidence for some results. In the present paper, a widely accepted index of overlap, the Corrected Covered Area index [8], based on a 50 % sample of reviews, was found to be in the low – moderate range.

The protocol of our study was not registered or published. However, since the approach followed was fixed during the study, this would not be expected to affect our results.

## Conclusions

This meta-review supports the conclusion that being a CG for PWD is a risk factor for psychological stress and physical ill-health. In addition, benefits in terms of psychological measures, such as mental health and depression, were generally found for CG coping strategies involving problem focus, acceptance and social-emotional support. However, negative outcomes for CGs were associated with wishful thinking, denial and avoidance coping strategies.

The interventions addressed in the reviews were extremely varied but could be grouped into Psychosocial, Psychoeducational, Technical, Therapy, Support Groups and Multicomponent. Both Psychosocial and Psychoeducational interventions were beneficial for CGs and PWD. Support groups, Multicomponent interventions and Joint Engagements by both CGs and PWD were generally found to be beneficial.

It was notable that virtually all reviews addressed very general coping strategies for stress broadly considered, rather than in terms of specific remedies for specific sources of stress. Consideration of specific stressors and remedies would seem to be a useful area for development for future research.

## Ethics approval and consent to participate

Not applicable (literature review).

## Consent for publication

Not applicable.

## Availability of data and materials

Data are in Additional file 2, and the references to each included study are in the main paper.

## Additional files

Additional file 1: PRISMA checklist. This file is a PRISMA 2009 Checklist for the current study. PRISMA stands for Preferred Reporting Items for Systematic Reviews and Meta-Analyses. It is an evidence-based minimum set of items for reporting in systematic reviews and meta-analyses. The checklist provides an indication of where items expected to be in a systematic literature review can be found in the text of the main paper. (DOC 62 kb) Additional file 2: Table of characteristics of included systematic reviews in meta-analysis. This file shows AMSTAR quality rating, authorship, year of publication, the focus of each review, the search strategy used, whether or not a meta-analysis was conducted, the number of papers included and excluded in each review, and the conclusions drawn from each review. (DOCX 531 kb)

## Abbreviations

AD: Alzheimer’s disease
ADL: activities of daily living
AMSTAR: assessment of multiple systematic reviews
BPSD: behavioural and psychological symptoms of dementia
CBT: cognitive behavioural therapy
CG: caregiver
MMSE: mini-mental state examination
PWD: people with dementia
QoL: quality of life
RCT: randomized controlled trial
