# Supplementary material for: A meta-review of stress, coping and interventions in dementia and dementia caregiving
Source: BMC Geriatr. 2016 May 18;16:106. doi: 10.1186/s12877-016-0280-8 (PMC4872341; doi:10.1186/s12877-016-0280-8)
Supplement: Additional file 2: — Table of characteristics of included systematic reviews in meta-analysis. This file shows AMSTAR quality rating, authorship, year of publication, the focus of each review, the search strategy used, whether or not a meta-analysis was conducted, the number of papers included and excluded in each review, and the conclusions drawn from each review. (DOCX 531 kb) [file 12877_2016_280_MOESM2_ESM.docx]

**Supplementary Table 1. Papers included in meta-review of dementia caregiving, interventions, coping and stress 1988-2014.**

| **AMSTAR**  **Score / 11** | **Authors and date** | **Systematic Review Focus** | **Search Methods** | **Meta-analysis** | **N included** | **N excluded** | **Conclusions** |
| --- | --- | --- | --- | --- | --- | --- | --- |
| 7 | 35 Acton & Kang (2001) | To evaluate intervention strategies to help caregivers cope with CG burden. | Searched MEDLINE (1966-1990, CINAHL (1982-1999), PsycINFO (1966-1999), ERIC (1966-1999), SSI (1989-1999), SSA (1983-1999) for keywords *caregiver, caregiving, dementia* and *Alzheimer’s disease* | Yes | 24 | 22 | Multicomponent intervention affected CG, support groups, education, psychoeducation counselling and respite care did not. Suggested that “Burden” too global an outcome to be useful. |
| 9 | 9 Bates, Boote & Beverley, (2003) | Psychosocial interventions for people with milder dementing illness.(ie MMSE > 15 and < 23) | Search of 15 electronic databases + 10 grey literature sources + general internet search (Google). Lead researchers contacted. Search terms included dement*, Alzheimer*, psychosocial, cognitive therap*, behavio(u)r* therap*, reality orientation, exercise* , etc. No date restriction. |  | 3 | 3694 | Small Numbers of cases in primary sources reviewed (19-28.)  No effects found for counselling or procedural memory stimulation (practise on ADL, such as brushing teeth.)Reality orientation showed some positive effects on cognition as indicated by MMSE and these seemed to be maintained. |
| 4 | 22 Black & Almeida (2004) | To assess associations between Behavioral and Psychological Symptoms of Dementia (BPSD) and CG burden, CG depression and institutionalization. | Searched MEDLINE, PsycINFO, CINAHL, 1990-2001, for *dementia* AND *behavior* OR *Mental disorders*; *Caregivers* AND *depression*. |  | 32 | 656 | Pooled correlations indicated moderate associations between BPSD and CG burden, CG distress and CG depression. Limited data suggested CG variables more important than BPSD in predicting institutionalization. |
| 7 | 10 Brodaty & Arasaratnam (2012) | To assess effectiveness of community based non-pharmacological (psychosocial) interventions for dementia patient symptoms and CG  Distress. | Searched MEDLINE, Embase, PubMed, PsycINFO, Scopus, 1985-2010, for combinations of condition terms: *dementia, Alzheimer*,*  Care provider terms: *caregiver*, carer*,*  Intervention terms: *treatment*, therap*, counsel*, intervention*, support, support group*,, psychosocial, nonpharmacologic** and symptom terms:  *Behavioural and psychological symptoms of dementia, BPSD, abberant motor behaviour, aggression, anxiety, etc.* | Yes | 23 | 1642 | Psychosocial interventions effective in reducing behavioural and psychological symptoms in patients (moderate effect sizes) and reducing CG distress (small effect size but significant). |
| 7 | 2 Brodaty, Green & Koschera (2003) | Meta-analysis of psychosocial interventions for caregivers of people with dementia | Search MEDLINE (1985-2000), PsycInfo (1984-2000), Ageline (1985-2000), CINAHL (1985-2000), Cochrane Library (1991-2000), Embase (1998-2000). For *caregiver, carer, self-help groups, support groups, education, training, skills training, counselling, psychotherapy, intervention*, and *therapy* combined with *random allocation, control group, dementia, Alzheimers disease.* | Yes | 30 | 24 | Caregiver (CG) interventions had modest but significant benefits for CG knowledge, psychological morbidity and coping skills. The only intervention feature related to outcome was involvement of the patent as well as the CG. |
| 3 | 33 Cooke, McNally, Mulligan, Harrison & Newman, (2001). | Psychosocial interventions for carers of people with dementia | Search PsycLit (1970-2000); MEDLINE (1966-2000); ISI (1981-2000); Embase (1980-2000); Cochrane Library (2000-Issue 3), for *(dement* OR Alzheimer*); carer*, caregiv* OR supporter*; AND (trial*, intervention* OR program*)*. Also hand searching of reference lists. |  | 40 | 2211 | 67% of studies showed no effects on any outcomes. Social support alone or in combination with problem focused approaches showed some benefits for psychological well-being. Interventions to increase knowledge generally did so but without impact on well-being or burden Typically small sample sizes limited chances of uncovering effects. |
| 7 | 36 Chien, Chu, Guo…Chou (2011) | Assess effectiveness of support groups for CGs | Searched OVID, NCBI, Cochrane Library, MEDLINE, PubMed, PsycArticles, PsycINFO, ERIC, Ageline, CINAHL (1998-2009), for *dementi** or *Alzheimer’s disease*,  *Support** or *psychoeducation** or *education** or *training*, group** or *program*, caregiv* or carer**. | Yes | 30 | 146 | Support groups showed significant positive effect on caregiver’s psychological well being, depression and burden. |
| 8 | 17 Cooper, Balamurali & Livingston (2007). | To assess prevalence and correlates of anxiety in CGs of people with dementia. | Searched Allied & Complementary Medicine (1985-), British Nursing Index (1994-), CINAHL (R) (1982-), Embase (1974-), MEDLINE (1951-), PsycINFO (1887-), up to June 2005, for  *Carer* OR *caregiver*, AND *dementia* OR *Alzheimer’s disease* AND *anxiety*. |  | 33 | 497 | C. 25% of CGs affected by significant levels of anxiety. Confrontative and escape avoidance coping, CG burden, poor CG health associated with higher anxiety levels. |
| 6 | 36 Cooper, Balamurali, Selwood & Livingston (2007) | Synthesise information re interventions to reduce CG anxiety. | Search MEDLINE, Embase, PsycINFO, CINAHL(R), British Nursing Index, Allied and Complementary Medicine. |  | 24 | 506 | Few interventions showed efficacy re anxiety. The only RCT to be effective involved CBT and a relaxation based intervention specifically devised for anxiety. Some evidence (not from RCTs) for benefits of yoga and relaxation; nut not for behavioural management, exercise or respite. |
| 9 | 37 Cooper, Mukadam, Katona, Lyketsos *et al* (2012) | To assess effectiveness of non-pharmacological interventions in improving QOL of people with dementia. | Search PubMed, Web of Science, Cochrane systematic reviews databases to January 2011, for *Dementia* OR *Alzheimer*; *Quality of life* OR *well-being*; AND *treatment* OR *intervention*. | Yes | 20 | 1049 | Coping-strategy based family carer therapy, with or without patient involvement, improved QoL of dementia patients living at home |
| 4 | 18 Cuijpers (2005). | To assess prevalence of depressive disorders in CGs of PWD | Searched Medline and Psychinfo up to May 2004 for *caregiv** AND *depression* |  | 10 | Not given | Overall prevalence of depressive disorder 22%. Relative risks ranged from 2.8 to 38.68 (all significant).Being a CG for PWD a risk factor for depressive disorder. |
| 5 | 32 Del-Pino-Casado, Frias-Osuna, Palomino-Moral & Pancorbo-Hidalgo,( 2011). | Caregivers coping and subjective burden | Search MEDLINE, Pubmed, CINAHL, EBSCO, EMBASE, Elsevier, PsycInfo, Ovid, Scopus, ISI Proceedings up to 2010 for *(caregiv* or supporter* or carer*) and (burden or strain) and (coping or cope)*. Also hand searching of journals, reference lists. |  | 10 | 687 | Avoidance coping associated with greater subjective burden.  No consistent pattern between problem focused, approach and emotion based strategies and burden emerged. |
| 6 | 38 Elvish, Lever, Johnstone, Cawley & Keady, (2013) | Psychological interventions for carers of people with dementia.  Updates previous reviews by Pinquart & Sorensen,(2006) and Gallagher-Thompson & Coon (2007). | Quantitative and qualitative lit search of MEDLINE, PsycInfo, ERIC and PubMed 2005-2011, for *(caregiver or caregiving or care or caring) AND (dementia or Alzheimer’s or cognitively impaired or cognitive impairment or mild cognitive impairment or MCI) AND (intervention study or evaluation study or experimental design or quasi-experimental design or memory clinic or counselling or psychoeducation or technology or support group or psychotherapy or case management or care management or multicomponent or environmental or cognitive behavioural therapy or CBT or family or systemic)*. |  | 20 | 1093 | Psychoeducational skill building to increase knowledge of AD and emotional coping was generally beneficial in maintaining well being. Multicomponent interventions (eg support groups, individual counselling, telephone support) and technology based approaches were useful. |
| 3 | 39 Etters, Goodall & Harrison (2008). | To identify factors influencing caregiver burden (CB) and describe evidence-based interventions to reduce CB | Searched CINAHL, MEDLINE, and PsychInfo (1966-2006) with keywords *CB* and *dementia* |  | 58 | N/A | Individually developed multicomponent interventions decrease CG burden, improve QoL and delay institutionalisation. |
| 4 | 27 Gottlieb & Wolfe,(2002). | Caregivers stress & coping | Search Current Contents, PsycInfo, Sociological Abstracts, Social Science Abstracts, (1984-2000), for *coping, eldercare, caregiving, carer, dementia, Alzheimer’s disease, older adults, caregiver stress.* |  | 17 | NA | Wishfulness & fantasy coping had negative impact; problem solving, acceptance and social support based coping were beneficial. |
| 7 | 40 Hall & Skelton (2011). | To identify evidence re current role of OTs in supporting caregivers of people with dementia (UK only). | Searched CINAHL, MEDLINE, Amed, PsychArticles, PsycInfo, ASSIA, Social Services Abstracts, Cochrane Library for *caregiver*, *dementia*, *occupational therapy*, *intervention* and *treatment*, (1999-2010). Hand searching also done. |  | 17 | 6 | Cognitive behavioural and reminiscence therapy can be effective re outcomes for CGs on eg., CG burden, stress, strain, health and mood. |
| 7 | 11 Hogan, Bailey, Carswell *et al.* (2007) | Management of mild to moderate AD: (ie MMSE >10 and < 26).  28 evidence based recommendations.  Focus on non-pharmacological recommendations. | Searched PubMed and Embase, for *dementia* OR *Alzheimer’s disease* AND *mild* OR *moderate* AND *therapy* OR *treatment*., 1996-2006. |  | 305 | 1310 | Insufficient evidence that cognitive training/rehabilitation is effective.  Individualised exercise programmes helpful.  Behavioural problems aided by support groups, psychoeducational intervention.  Involvement of CG and CR in intervention helps. |
| 4 | 28 Kneebone & Martin, (2003). | Caregivers stress & coping | Search MEDLINE, Psych-Info to 1999 for *Alzheimer’s disease, dementia, caregivers burden, adaptation, psychological, coping, stress* |  | 16 | N/A | Applying Lazarus & Folkman’s 1984 model, found benefits re health and depression from problem solving and acceptance styles of coping. |
| 4 | 1 Knight, Lutzky & Macofsky-Urban (1993) | Assess effectiveness of interventions for CG distress. | Searched relevant journals and Ageline, MEDLINE, PsycINFO and SSI, 1980-1990.  Keywords not given. | Yes |  | na | Individual psychosocial interventions and respite programs moderately effective; Group psychosocial interventions less so. |
| 5 | 21 Lee, Bakker, Duivenvoorden & Droes (2014) | To assess determinants of subjective CG burden in dementia | Searched PubMed, PsycInfo and Embase up to December 2013 for *burden, distress, stress, strain, overload, well-being* AND *caregiver, family, spouse, informal, relative, home* AND *psychogeriatrics, geriatric psychiatry, dementia, behaviour, neuropsychiatric symptoms* OR *theoretical, concept, predictors, prognostics, model, scale, limited by (old) age and human.* |  | 56 | 711 | Patient behavioural problems, caregiver coping and personality traits and competence were most consistent determinants of CG burden, depression and mental health. Behavioural problems were more significant than cognitive disorders or lack of self-care. Of CG personality traits, neuroticism had strongest impact on burden. CG feeling competent or higher self-efficacy were beneficial re burden and mental health. |
| 8 | 30 Li, Cooper, Austin & Livingston (2013) | Do changes in coping style explain effectiveness of interventions for dementia carers? | Searched Embase, MEDLINE, PsycINFO, Web of Science, Cochrane Library, CINAHL and AMED, up to July 2011, for *carer* OR *caregiver* OR *caring* OR *relative* OR *supporter* OR *family*; *dementia* OR *Alzheimer* OR *cognitive impairment*; *cope* OR *coping*; AND *randomised* OR *controlled* OR *clinical trial*. | Yes | 8 | 425 | Surprisingly, dysfunctional coping increased when depressive symptoms declined. Some evidence that emotional support and acceptance based coping increased as positive coping increased, but solution focused coping alone did not. |
| 7 | 31 Li, Cooper, Bradley, Schulman & Livingston (2012). | Caregivers coping & psychological morbidity | Searched Embase, MEDLINE, PsycInfo, Web of Science, CINAHL, AMED to March 2010, for *(carer OR caregiver OR caring OR relative OR supporter OR family); (dementia OR Alzheimer OR cognitive impairment ); coping; (anxiety OR depression OR mood OR psychiatric morbidity OR psychological morbidity)*. Also hand search reviews & references. | Yes | 33 | 5361 | Dysfunctional coping (eg denial/avoidance) associated with greater depression & anxiety.  Emotional support and acceptance based coping associated with less anxiety and depression.  Solution focused coping not associated with depression or anxiety. |
| 2 | 42 LoGiudice & Hassett, (2005) | Caregivers & uncommon dementias (FTD, HD, HIV) | No search strategy stated. |  | 13 | na | Disturbed behaviours typical of uncommon dementias associated with increased depression in carers. |
| 4 | 43 Luppa, Luck, Brahler, Konig & Riedel-Heller (2008) | To assess factors influencing institutionalisation of persons with dementia. | Searched MEDLINE, Web of Science, Cochrane Library and PSYNDEX for keywords, *Institutionalisation, Nursing home placement, nursing home admission and dementia.* |  | 42 | 467 | CG burden , life satisfaction, health related QoL and dysfunctional coping strategies linked to earlier institutionalization |
| 8 | 12 O’Connor, Ames, Gardner & King (2009). | To review psychosocial treatments of behaviour symptoms in AD | Searched MEDLINE, CINAHL, PsycINFO, Cochrane databases up to 2006.  Keywords not given. |  | 25 | 93 | Effect sizes v controls mostly small; moderate to large effects with aromatherapy, ability focused career education, bed baths, music and muscle relaxation training. |
| 5 | 13 Olazaran, Reisberg, Clare, Cruz…..Muniz (2010) | To evaluate effects of Nonpharmacological Therapies on Quality of Life of PWD and CGs of PWD. | Searched MEDLINE, PsycINFO, CINAHL, Embase, Lilacs, Cochrane Dementia and Cognitive Improvement Group Specialized Register, up to September, 2008.  Keywords not given in paper. | Yes | 179 | 1134 | Strong evidence that Multicomponent interventions for CGs education and support delayed institutionalisation.  Some evidence for improvements linked to multicomponent interventions for PWD cognition, Activities of Daily Living, behaviour & mood, and for CG mood, well-being and Quality of Life. |
| 6 | 25 Ornstein & Gaugler (2012) | To determine whether particular symptoms or symptom clusters exert undue negative impact on CG depression and burden. | Searched MEDLINE, Pubmed and PsycINFO for *dementia* OR *Alzheimer*, *caregivers* OR *caregiving*, *behaviour symptom* OR *BPSD* OR *psychiatric* OR *neuropsychiatric* OR *hallucination* OR *delusion* OR *aggression* OR *agitation* OR *wandering* OR *psychosis* OR *depression* OR *behaviour*. |  | 35 | 673 | Depression, aggression and sleep deprivation were most frequently identified patient symptoms to impact negatively on CGs. However, overall, a wide range of symptoms was associated with CG depression and burden. |
| 7 | 44 Parker, Mills & Abbey (2008) | To assess effectiveness of interventions to assist CGs to provide support for dementia patients in the community. | Extensive search of CINAHL, MEDLINE, PsycINFO and Cochrane databases. |  | 40 | 645 | 12/13 psychoeducational interventions had positive results for depression and burden. Studies of support showed a small but significant benefit for burden.  10/12 multicomponent studies reported significantly improved outcomes. |
| 7 | 45 Peacock & Forbes (2003) | To assess interventions to improve well being of CGs | Searched CINHAHL, Pubmed, PsycINFO 1992-2002, for keywords *caregiver, carer, Dementia, Alzheimer, burden, depression, strain, stress, support, respite, education, intervention, effective, assess, evaluate and measure.*  Also hand searched relevant journals. |  | 11 | 81 | None of the types of interventions (case management, education, psychotherapy, computer networking) had consistent effects overall. |
| 7 | 34 Pinquart & Sorensen (2006) | Which interventions for caregivers of AD patients help and how much? | Searched PsycINFO, MEDLINEe, Ageline, PSYNDEX (dates not specified), for (*dementia* OR *Alzheimer’s disease*) AND (*caregiver* OR *care* OR *caregiving*) AND (*intervention* OR *trial* OR *support* OR *training*).  Note: overlaps strongly with Brodaty *et al* (2003) but extends coverage to more recent studies. | Yes | 127 | No info | CG interventions had positive immediate effects on CG’s burden, depression, well being, ability/knowledge, and CR symptoms. Effect sizes small. Psychoeducational interventions had broadest effects and were most effective when involved active participation. |
| 7 | 19 Pinquart & Sorensen (2007). | To assess correlates of physical health of informal CGs | Searched PsycINFO, MEDLINE, PSYNDEX for keywords *health* AND (*caregiving* OR *Caregivers* OR *carer* OR *support provider*) AND (*elderly* OR *old age*) |  | 176 | Not given | Negative effects on CG health most common in in psychologically distressed CGs facing dementia –related stressors. |
| 4 | 46 Powell, Chiu & Eysenbach (2008). | To assess effectiveness of networked ICT interventions in supporting CGs of people wirh dementia. | Searched MEDLINE, Embase, CINAHL, PsycINFO, AMED up to 2007, for *dementia, Alzheimer’s, carer, caring,* AND *network, internet.* |  | 15 | 1441 | Interventions were multifaceted with inconsistent outcomes, but interventions tended to have moderate effects on CG stress and depression. |
| 7 | 29 Pusey & Richards (2001) | Assess effectiveness of psychosocial interventions for CGs | Searched MEDLINE, CINAHL, Embase, Cochrane Library, HMIC, SCI, SSCI, Age Info, National Research Register and Health CD. Plus hand searching of relevant journals. Keywords not given. |  | 30 | 361 | Study quality generally poor. Individualised interventions stressing problem solving and behaviour management most effective. |
| 5 | 47 Quinn, Clare & Woods (2010) | To explore impact of meaning and motivation on well being of CGs | Searched, PsycINFO, SSCI, MEDLINE and CINAHL, 1960-2008, for keywords *caregiver*, carer*, caregiving, dementia, Alzheimer’s, motiv*, drive*, oblig*, duty, filial* and *meaning** |  | 10 | 2 | CGs’ well being affected by nature of motivations. Finding meaning had a positive impact on CGs’ well being. |
| 8 | 48 Schoenmakers, Buntinx & deLepeleire, (2010). | Effects of home care intervention on carer well being | Search of MEDLINE, Embase, Cochrane Database of Systematic Reviews, Database of Abstracts of reviews of Effects , Cochrane Register of Controlled Trials and ACP Journal Club, (1980-2007), for *dementia, primary caregiver, interventions , home care* and their abbreviated terms. | Yes | 26 | 880 | On a meta-analysis, Psychosocial intervention had small positive but non significant effects on depression and burden. |
| 4 | 49 Schulz, O’Brien, Czaja, Ory….Stevens (2002) | To assess clinical significance of caregiver interventions. | Searched MEDLINE, PsycINFO, CINAHL, (1996-2001), for *caregivers* and *dementia* or *Alzheimer’s disease*. |  | 50 | na | Relatively few studies showed clinically significant effects so far but conclude that interventions are promising and may show up more strongly with improved methods. |
| 6 | 50 Selwood, Johnston, Katona, Lyketsos & Livingston (2007). | To identify effective psychological interventions for CGs | Databases searched and keywords not reported.  Search went up to 2003. |  | 62 | 182 | Individual behaviour management therapy re patients’ behaviour effective. Teach caregivers coping strategies also effective. Education re dementia, group behaviour therapy and supportive therapy not effective. |
| 6 | 16 Smits, de Lange, Droes…Pot (2007) | Assess evidence for benefits of combined intervention programmes (ie that address both the patient and the CG) for patient and CG. | Search MEDLINE and Psychinfo 1992-2005., for *dementia, family members, caregivers, caregiver burden, support programme, training, counselling, care-giving skills, intervention, combined intervention, integrated intervention, effect*, effic** |  | 25 | 27 | Best results were for CG general mental health. Effects on cognitive, physical functioning, behaviour problems and survival of patents were small and inconsistent, although their mental health and time to admittance to long term care were positively affected. |
| 7 | 51 Sorensen, Pinquart & Duberstein, P. (2002) | Determine effectiveness of interventions for CGs | Searched PsycINFO, MEDLINE, PSYNDEX, for *caregiver* or *carer* or *caregiving*, *intervention* or *support* or *training*, and *elderly* or *old age*. | Yes | 78 | 61 | Interventions generally successful.  Effects larger on ability/knowledge than on burden/depression.  Psychoeducational/ therapeutic effects most consistent overall. |
| 7 | 14 Spijker, Vernooij-Dassen, Vasse, Adang…Verhey (2008). | Assess effectiveness of support programs in delaying institutionalisation of AD patients | Searched MEDLINE, Web of Knowledge, PsycINFO, (January 1990-March 2006), for *controlled studies, dementia costs, institutionalisation, time spent giving care, caregivers* | Yes | 13 | 442 | Support programmes significantly decreased odds of institutionalisation and lengthened time to institutionalisation.  Actively involving CGs in choices about treatments was key to programme effectiveness. |
| 4 | 15 Thinnes & Padilla (2011) | To review evidence re effectiveness of educational and supportive strategies for CGs | See Arbesman & Leiberman (2011) for method |  | 43 | 7402 | Interventions that jointly engage patients and CGs in education and training more effective. Interventions providing CGs with problem solving, technical skills, home modification and referral to community resources are useful. |
| 8 | 52 Thompson, Spilsbury, Hall, Birks, Barnes & Adamson,J. (2007). | To examine whether information and support interventions imppove Quality of Life for CGs | Searched Specialized Register of Cochrane Dementia and Cognitive Improvement Group 2003-05 for *computer*, telephon*, training*, education*, information, “care-planning”, carer*, caregiv** | Yes | 44 | 97 | Group based interventions had positive impact on psychological morbidity; technology and individual based interventions not effective. |
| 8 | 53 Vernooij-Dassen, Draskovic, McCleery & Downs (2011). | To evaluate cognitive reframing interventions for CG’s psychological morbidity and stress | Searched Cochrane Library, MEDLINE, Embase, PsycINFO , CINAHL and LILACs, using extensive keyword lists. | Partly | 11 | 73 | Cognitive reframing reduced psychological morbidity, specifically anxiety, depression and stress. |
| 4 | 20 Vitaliano, Zhang & Scanlan (2003). | To assess physical health risks for CGs of PWD. | Searched Current Contents, MEDLINE, PsycINFO, Sociofile, Social Work Abstracts, and CINAHL to April, 2001, using keywords *dementia, Alzheimer’s disease, cognitive disorders, health, physical* and *health, physiology*, illness*, hormones, cholesterol, cardiovascular diseases, blood pressure, obesity, diabetes, immune*, mortality, death* and *caregiv** | Yes | 23 | Not reported | Evidence found of links between being a CG of PWD v controls and level of stress hormones (23% higher in CGs), antibodies (15 % lower in CGs) and global reported health. |
| 9 | 3 Zabalegui, Hamers, Karlsson ,….Cabrera (2014). | To identify effective interventions to improve quality of care | Searched MEDLINE, CINAHL, PsycINFO and ISI Web of Science, 1990-2012 for principal keywords *dementia*, *Alzheimers disease*, *patient care* and *home care* |  | 23 | 743 | Cognitive rehabilitation effective at early stages of AD. Case managers reduce institutionalisation and use of other services. |
